# Hypoxia regulates Toll-like receptor-9 expression and invasive function in human brain cancer cells in vitro

**DOI:** 10.3892/ol.2014.2095

**Published:** 2014-04-25

**Authors:** JOUKO SANDHOLM, JOHANNA TUOMELA, JOONAS H. KAUPPILA, KEVIN W. HARRIS, DAVID GRAVES, KATRI S. SELANDER

**Affiliations:** 1 Department of Medicine, University of Alabama at Birmingham, Birmingham, AL 35294, USA; 2 Department of Pathology, Oulu University Hospital, Oulu 90029, Finland; 3 Department of Surgery, Oulu University Hospital, Oulu 90029, Finland; 4 Department of Anatomy and Cell Biology, University of Oulu, Oulu 90570, Finland; 5 Birmingham Veterans Affairs Medical Center, Birmingham, AL 35233, USA; 6 Department of Chemistry, University of Alabama at Birmingham, Birmingham, AL 35294, USA

**Keywords:** Toll-like receptor-9, hypoxia, invasion, brain cancer

## Abstract

Toll-like receptor-9 (TLR9) is a cellular DNA sensor of the innate immune system. TLR9 is widely expressed in a number of tumors, including brain cancer; however, little is known regarding its regulation and involvement in cancer pathophysiology. The present study demonstrated that hypoxia upregulates and downregulates TLR9 expression in human brain cancer cells *in vitro*, in a cell-specific manner. In addition, hypoxia-induced TLR9 upregulation was associated with hypoxia-induced invasion; however, such invasion was not detected in cells where hypoxia had suppressed TLR9 expression. Furthermore, suppression of TLR9 expression through TLR9 siRNA resulted in an upregulation of matrix metalloproteinase (MMP)-2, -9 and -13 and tissue inhibitor of matrix metalloproteinases-3 (TIMP-3) mRNA, and a decreased invasion of cells in normoxia, in a cell-specific manner. In cells where hypoxia induced TLR9 expression, TLR9 expression and invasion were reduced by TLR9 siRNA. The decreased invasion observed in hypoxia was associated with the decreased expression of the MMPs and a concomitant increase in TIMP-3 expression. In conclusion, hypoxia regulates the invasion of brain cancer cells *in vitro* in a TLR9-dependent manner, which is considered to be associated with a complex expression pattern of TLR9-regulated mediators and inhibitors of invasion.

## Introduction

The Toll-like receptors (TLRs) are key regulators of the innate immune system and function as pattern recognition proteins that detect microbe- and host-derived endogenous molecular patterns (1,2). Thus far, 13 mammalian TLRs have been identified and each TLR recognizes a specific ligand; TLR4 is the receptor for bacterial lipopolysaccharide, TLR5 recognizes flagellin and members of the TLR9 subfamily recognize RNA (TLR3, 7 and 8) and DNA (TLR9) structures (1,2). Differences have also been identified in the subcellular localization of the various TLRs; TLR1, 2 and 4 are typically cell surface bound, whereas the nucleotide-sensing receptors, TLR3 and TLR13, as well as the TLR9 subfamily, reside in the intracellular compartments (3–6). Ligand binding to TLRs activates transcription factors, most notably NF-κB and the eventual outcome of TLR activation in cells of the immune system is an innate immune reaction, which is characterized by increased cytokine and interleukin production (2). Eventually, this inflammatory reaction results in the activation of the adaptive immune system and thereby, elimination of the invading pathogens and infected cells (7).

It has been well established that in addition to the cells of the immune system, TLR9 is frequently expressed in various cancer cell lines, as well as in clinical cancer specimens (5,8–16). Furthermore, synthetic TLR9-ligands (CpG-sequence containing oligonucleotides) have been demonstrated to stimulate the *in vitro* invasion of TLR9-expressing cancer cells, including astrocytoma and glioblastoma cells (14,16). This increased invasion is mediated via the downregulation of the tissue inhibitor of matrix metalloproteinases-3 (TIMP-3) and the upregulation of matrix metalloproteinase-13 (MMP-13) activity (15,16). Additionally, increased TLR9 expression has been associated with the poor differentiation of cancer cells in breast, prostate and glioblastoma multiforme (GBM) tumors (17–21). Furthermore, in GBM patients, high expression of the TLR9 protein in tumors has been found to correlate with a significantly shorter survival time (17). Despite the well-documented expression of TLR9 in various cancers and invasive response to TLR9 ligands *in vitro*, the involvement of this protein in cancer pathophysiology remains unclear. The regulation of TLR9 expression, as well as the possible physiological ligands which may induce invasion in TLR9-expressing cancer cells, also remain poorly characterized. Synthetic TLR9 ligands have also been investigated in preclinical models of brain cancer immunotherapy; CpG-sequence containing oligonucleotides were shown to induce apoptosis in brain cancer cells *in vitro* and *in vivo* and, therefore, may provide long-term antitumor immunity against gliomas (22,23).

Hypoxia is a fundamental characteristic of solid tumors and it induces various adaptive changes in cancer cells, which eventually lead to increased tumor growth, invasion and metastasis (24). Hypoxia is also frequently detected in brain tumors and the detection of hypoxia in these tumors by hypoxia markers is essential for the diagnosis of GBM (25). Due to the previously demonstrated associations between hypoxic tissue conditions and the upregulation of TLR2 and 6 in various normal tissues, we hypothesized that hypoxic tissue conditions may activate TLR9-expression and the TLR9-mediated invasive pathway in brain cancer cells (26).

## Materials and methods

### Cell culture

Human D54MG, U373MG and SNB191 glioma cell lines and primary human GBM XD45 and JX10 xenolines (UAB Brain Tumor Specialized Program Of Research Excellence) were cultured in Dulbecco’s modified Eagle’s medium (Gibco-BRL, Paisley, UK) supplemented with 10% heat-inactivated fetal bovine serum, L-glutamine, penicillin/streptomycin and non-essential amino acids (all Gibco-BRL) (27,28). The cells were cultured at 37°C, in a humidified atmosphere of 5% CO_2_ and 95% air (~21% pO_2_). For the hypoxia experiments, the cells were kept for the indicated durations in a cell culture incubator (I-Glove; BioSpherix, Inc., Lacona, NY, USA) with an oxygen level set to 1 or 5% pO_2_, as indicated. Chloroquine was purchased from Sigma-Aldrich (St. Louis, MO, USA) and the wide-spectrum MMP-inhibitor, GM6001, was obtained from Enzo Life Sciences Inc., (Farmingdale, NY, USA).

### RNA isolation and quantitative polymerase chain reaction (qPCR)

Total RNA was isolated from the cells using TRIzol reagent (Invitrogen Life Technologies, Carlsbad, CA, USA) and purified using RNeasy mini kits (Qiagen, Valencia, CA, USA). All reagents used for the qPCR experiments were purchased from Applied Biosystems (Carlsbad, CA, USA). The cDNA was synthesized from 0.2 μg of total RNA, using MultiScribe reverse transcriptase and random hexamers. TLR9 mRNA expression levels were then quantified using the following primer and probe set, obtained from Applied Biosystems: Forward, 5′-GGCCCTCCACGCATGAG-3′ and reverse, 5′-CTTGTCCTTTTCTGCCCTTGTAG-3′ for TLR9; and 5′-CCTGCAGAACTCTG-3′ for the probe. The primer and probe sets used for MMP-2, MMP-9, MMP-13 and TIMP-3 were also purchased from Applied Biosystems. For all qPCR assays, a standard amplification program was used as follows: One cycle of 50°C for 2 min; one cycle of 95°C for 10 min; and 40 cycles of 95°C for 15 sec and 60°C for 1 min. Following normalization to the large ribosomal protein RPLPO expression levels for each cDNA, a relative quantification of the target cDNA was performed using 2^−ΔΔCt^ values (29).

### Western blot analysis

The cells were cultured in normal culture medium until near confluency and then rinsed with sterile phosphate-buffered saline (PBS; Fisher Scientific, Pittsburgh, PA, USA), prior to culture in serum-free culture medium (Gibco-BRL). After 24 h, the culture medium was discarded and the cells were rapidly harvested in lysis buffer (Cell Signaling Technology, Inc., Danvers, MA, USA), prior to separation by centrifugation as previously described (30). Next, the supernatants were boiled in reducing SDS sample buffer and equal amounts of protein (~100 μg) were loaded into each lane. The samples were then electrophoresed into 10% or 4–20% gradient polyacrylamide SDS gels (Bio-Rad, Hercules, CA, USA) and transferred to nitrocellulose membranes (Bio-Rad). Following blocking with 5% non-fat dry milk in Tris-buffered saline with Tween-20 (TBST), the blots were incubated overnight at 4°C with the following primary antibodies: TLR9 (IMG-431; 1:500, Imgenex Corporation, San Diego, CA, USA), TIMP-3 (AB8106; 1:500, Millipore, Billerica, MA, USA) and actin (A-2066; 1:1,000, Sigma-Aldrich, St. Louis, MO, USA). All primary antibodies were diluted in 0.1% TBST (v/v), with 5% non-fat dry milk. The secondary detection was performed using the horseradish-peroxidase-linked secondary antibodies (NA934-100UL, Anaspec, Fremont, CA, USA) and the protein bands were visualized with Pierce Enhanced Chemiluminescence Western Blotting substrate (Pierce Biotechnology, Inc., Rockford, IL, USA) (30).

### Zymograms

The cells were cultured on 12-well plates until confluent, and then washed with sterile PBS and further cultured for 24 h in serum-free media (500 μl per well). The supernatants were then collected and concentrated using a centrifugal filter device using a cut-off size of 3 kDa (UFC5-003-24; Millipore, Billerica, MA, USA). An equal amount of protein (~20 μg) was added to each lane of the zymogram gels (10% gelatin; Bio-Rad). The gels were then run, renaturated and developed using BioRad zymogram buffers, according to the manufacturer’s instructions (31).

### RNA interference

Downregulation of TLR9 with siRNA was performed using a plasmid-based approach and a previously described TLR9 or control (non-targeting siRNA) siRNA sequence, which were cloned into the pSuper-EGFP vector (Oligoengine, Seattle, WA, USA) (14,31). The plasmids were stably transfected into the cells using standard techniques (14). The G418 (0.8 mg/ml) selection was maintained and four cycles of green fluorescence-based cell sorting was performed to obtain a pool of cells with a high percentage of green fluorescent protein positivity. For the U373MG cells, oligonucleotide siRNA molecules, the human TLR9 siRNA Smart pool or control human non-targeting siRNA pool (Dharmacon, Inc., Lafayett, CO, USA) were used. Briefly, 8 μl of siRNA stock (20 μM) was added to 500 μl Optimem (Gibco-BRL, Grand Island, NY, USA) on six-well plates, followed by the addition of 5 μl Lipofectamine RNAimax (Invitrogen Life Technologies, Grand Island, NY, USA). The components were mixed by pipetting and the plates were agitated on a platform for 20 min at room temperature, followed by the addition of 0.8 ml of cell suspension (45×10^4^ cells/ml). After 2 h, 1.6 ml of regular growth medium was added to the cells and the medium was changed on the following day. After 48 h, the cells were trypsinized and subsequently used in invasion assays or cultured in serum-free media for the final 24 h in normoxic or hypoxic conditions, prior to western blot analysis.

### Invasion assays

Invasion assays were performed as previously described (16). Briefly, the cells (20,000 per insert) were allowed to invade the extracellular matrix-like Matrigel (BD Biosciences, Franklin Lakes, NJ, USA) in 24-well plate inserts (Becton-Dickinson, Franklin Lakes, NJ, USA). The membranes were stained using the Hema-3 set (Thermo Fisher Scientific, Rockford, IL, USA) and the number of invading cells was counted under a microscope (Nikon Labophot-2, Nikon Instech Co., Ltd., Tokyo, Japan) (16). In certain experiments, a 22-mer DNA-oligonucleotide, which forms G-quadruplex structures (sequence, aggg tta ggg tta ggc taa ggg in a phosphodiester backbone; Midland Certified Reagent Company, Inc., Midland, TX, USA), was used as a positive control at a final concentration of 10 μM and added to the upper invasion wells. Through our ongoing studies we have determined that this DNA-ligand induces TLR9-mediated invasion (32).

### Cell viability assays

In total, 20,000 cells were plated on 96-well plates (100 μl/well) in normal growth medium. The viability of the cells was measured using the CellTiter 96 AQueous One Solution Cell Proliferation assay (Promega, Madison, WI, USA), according to the manufacturer’s instructions. In an additional set of assays, the cells were plated on 24-well plates and, after the indicated time, the cells were trypsinized and the viable cells were counted using the TC10™ automated cell counter (Bio-Rad).

### Statistical analysis

Data are presented as the mean ± standard deviation or mean ± standard error of the mean, as stated. Student’s t-test was used to determine whether the differences between the corresponding cells under various circumstances were statistically significant. P<0.05 was considered to indicate a statistically significant difference.

## Results

### Hypoxia induces TLR9 expression in human D54MG and U373MG cells

**Figure 1:**
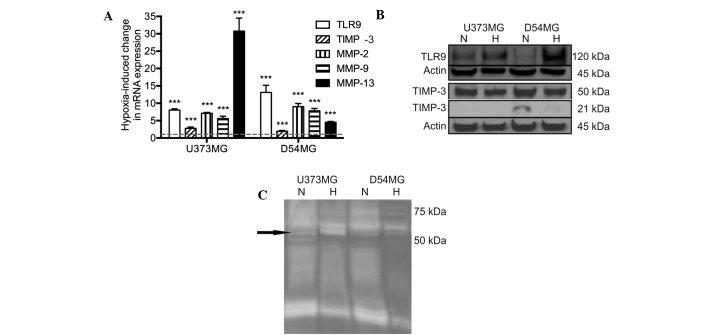
(A) U373MG and D54MG cells were cultured for 24 h under normoxia and hypoxia. mRNA expression was then measured using quantitative polymerase chain reaction. Bars present the hypoxia-induced changes, relative to normoxia (indicated by the dotted line). Data are presented as the mean ± SD (n= 6) ^***^P<0.001, vs. the corresponding value in normoxia. (B) Western blot analysis of TLR9 and TIMP-3 in U373MG and D54MG cells cultured for 24 h under normoxia and hypoxia. Actin bands were used to indicate equal loading. (C) Zymograms demonstrating the proteolytic activity of conditioned medium of U373MG and D54MG cells. Arrow indicates the activity of active MMP-2 in normoxia and hypoxia. TLR9, toll-like receptor-9; TIMP-3, tissue inhibitor of matrix metalloproteinases-3; MMP, matrix metalloproteinase; N, normoxia; H, hypoxia.

The D54MG and U373MG cells were selected for the current study as they have been demonstrated to express TLR9 and exhibit increased invasion *in vitro* in response to synthetic TLR9-ligands (16). Furthermore, brain cancers have been shown to be highly associated with the tumor markers of hypoxia. In the current study, the cells were cultured under normoxic and hypoxic conditions for 24 h and the mRNA expression was determined using qPCR. Hypoxia was found to induce a significant upregulation of TLR9 mRNA expression in the two cell lines. In addition, the mRNA expression levels of TIMP-3 and MMP-2, -9 and -13 were also found to significantly increase simultaneously (Fig. 1A). The protein expression levels of TLR9 were found to correlate with the mRNA levels in the two cell types. By contrast, the protein expression levels of TIMP-3 were found to marginally decrease in hypoxia in the D54MG and U373MG cells (Fig. 1B). The proteolytic activity of the conditioned medium of the U373MG and D54MG cells was analyzed using zymograms. A triple band of ~60 kDa was observed, which may present latent MMP-2 and active MMP-2 and MMP-13. Additionally, an increased level of MMP-2 activity in U373MG cells was detected in hypoxia when compared with normoxia (Fig. 1C).

### TLR9 regulates MMP and TIMP-3 expression in normoxia

**Figure 2:**
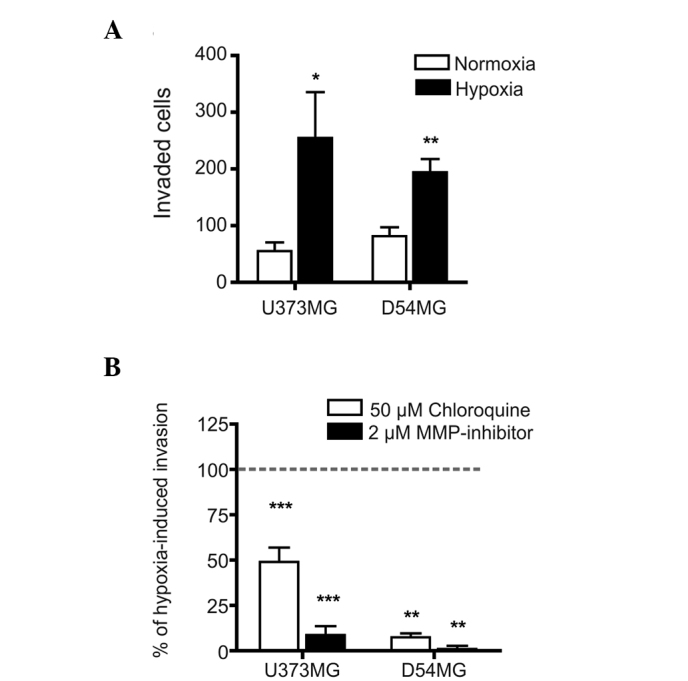
(A) Matrigel invasion assays of parental U373MG and D54MG cells cultured in normoxia or hypoxia. Data are presented as the mean ± SEM (n=6) ^*^P<0.05 and ^**^P<0.01, vs. the corresponding cells in normoxia. (B) Invasion assays of the same cells in hypoxia, with or without 50 μM chloroquine or 2 μM MMP inhibitor GM6001 for 24 h. Data are presented as the percentage of hypoxia-induced invasion (mean ± SD, n=3) ^**^P<0.01 and ^***^P<0.001, vs. the corresponding vehicle treatments. MMP, matrix metalloproteinase.

Hypoxia has been shown to induce invasion in various types of cancer cells (31,33,34) and, therefore, the involvement of TLR9 in the process of invasion was investigated. The invasive effects of hypoxia were first characterized in the parental U373MG and D54MG cells and, as expected, hypoxia induced a significant increase in the invasion rates of the two parental cell lines (Fig. 2A). Furthermore, the hypoxia-induced invasion of these cells was significantly inhibited by chloroquine, an important inhibitor of endosomal acidification (and, therefore, possibly TLR9 signaling) and almost completely blocked by a wide-spectrum of MMP-inhibitors (Fig. 2B). These results suggested that TLR9 signaling and MMP activity regulate hypoxia-induced invasion in these cells.

To study the involvement of TLR9 in this process more specifically, stable TLR9 siRNA cells were established, in which TLR9 expression was downregulated through a plasmid-based siRNA approach (31). In a similar manner, the control cells were transfected with a plasmid encoding a non-targeting siRNA. Characterization of the control and TLR9 siRNA cells in normoxic, steady-state conditions is shown in Table I. Briefly, relative to the corresponding control siRNA cells, the TLR9 siRNA cells exhibited downregulated TLR9 mRNA expression levels of ~37 and ~66% in the U373MG and D54MG cells, respectively. Furthermore, the downregulation of TLR9 induced cell-specific changes in cellular MMP and TIMP-3 mRNA expression levels. The mRNA expression levels for various MMPs (MMP-2, -9 and -13) remained unchanged or were marginally upregulated in the two cell lines; however, downregulation of TLR9 resulted in a significant upregulation of TIMP-3 mRNA expression (Table I.).

**Figure 3:**
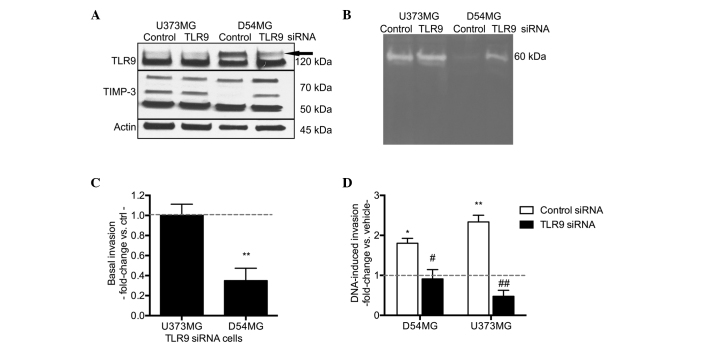
Characterization of the control and TLR9 siRNA cells in normoxia. (A) Expression of TLR9 (indicated by the arrow) and TIMP-3 proteins in the indicated cells. Actin bands were used to indicate equal loading. (B) Zymograms of the supernatants of the indicated cells cultured in normoxia. (C) The basal invasive capacity of the indicated TLR9 siRNA cells. Data are presented as the percentage of basal invasion of the corresponding control siRNA cells in normoxia (mean ± SEM; n=9–13) ^**^P<0.01, vs. the corresponding control siRNA cells (indicated by the dotted line). (D) TLR9-ligand (G-quadruplex-DNA) induced invasion in control and TLR9 siRNA cells. Data are presented as a fold-increase of invasion, as compared with the vehicle treatment (indicated by the dotted line; mean ± SEM; n=3–9) ^*^P<0.05 and ^**^P<0.01, vs. the corresponding vehicle-treatment; and ^#^P<0.05 and ^##^P<0.01, vs. the corresponding control siRNA cells. TLR9, toll-like receptor-9; TIMP-3, tissue inhibitor of matrix metalloproteinases.

At the protein level, the changes in TLR9 expression between the control and TLR9 siRNA cells in normoxia were subtle and similar to that of the mRNA expression (Fig. 3A). Consistent with the changes at the mRNA level, D54MG/TLR9 siRNA cells also exhibited increased TIMP-3 protein expression; however, this upregulation was particularly detected in TIMP-3 protein bands of high molecular weight, which may subsequently present different glycosylation forms of the protein (35,36). In the U373MG/TLR9 siRNA cells, TIMP-3 protein expression in normoxia was similar to that of the U373MG/control siRNA cells (Fig. 3A). A comparison of the proteolytic activity between the control and TLR9 siRNA supernatants in normoxia demonstrated differences between the control and TLR9 siRNA D54MG cells. More specifically, the supernatants of the D54MG/TLR9 siRNA cells exhibited enhancement in a 60-kDa proteolytic band, suggesting an increase in latent MMP-2 (Fig. 3B). No such differences were detected between the proteolytic activities of the U373MG control siRNA and TLR9 siRNA supernatants in normoxia. The basal invasive capacity of D54MG/TLR9 siRNA cells in normoxia was significantly decreased, as compared with the D54MG/control siRNA cells. However, no difference was detected between the control and TLR9 siRNA U373MG cells (Fig. 3C).

Finally, to further confirm the differences in TLR9-mediated invasion between the control and TLR9 siRNA cell pairs, the invasive capacity of these cells was also studied in response to treatment with G-quadruplex-DNA TLR9-ligands in normoxia. As compared with the vehicle, the G-quadruplex-DNA induced an approximately two-fold increase in invasion in the D54MG/control siRNA cells, but not in the corresponding TLR9 siRNA cells. Similar results were observed with the stable U373MG cells, confirming differences in the TLR9-mediated invasive responses of the TLR9 siRNA cells (Fig. 3D).

Overall, these results suggest that the downregulation of TLR9 in brain cancer cells results in decreased basal and DNA ligand-induced invasion in normoxia, in a cell-specific manner. The anti-invasive phenotype, particularly of the D54MG/TLR9 siRNA cells, suggests that despite the observed increases in MMP mRNA expression, the increased expression levels of the endogenous MMP-inhibitors, such as TIMP-3, are possibly a more significant determinant of their invasive behavior in normoxia.

### Hypoxia-induced invasion is inhibited in TLR9 siRNA brain cancer cells

**Figure 4:**
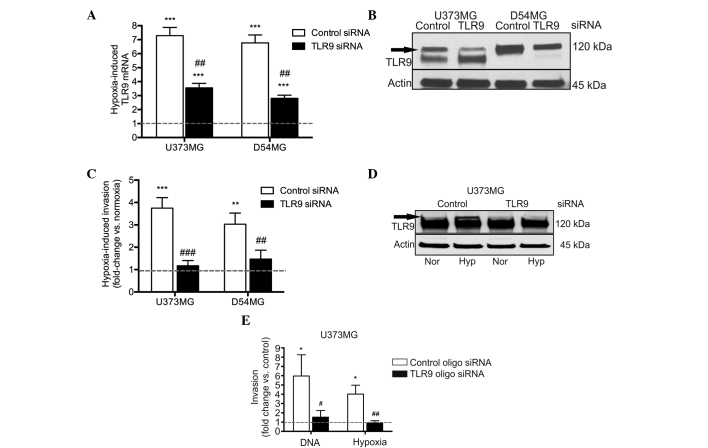
Hypoxia-induced TLR9 (A) mRNA and (B) protein expression as indicated by the arrow in the U373MG and D54MG cells. Actin bands were used to indicate equal loading. (C) Hypoxia-induced invasion in the indicated cells. Data are presented as a fold-increase of invasion in hypoxia, as compared with invasion in normoxia (indicated by the dotted line; mean ±SEM; n=7–12) ^**^P<0.01 and ^***^P< 0.001, vs. normoxia; and ^##^P<0.01 and ^###^P<0.001, vs. the corresponding control siRNA cells. (D) Expression of TLR9 protein (indicated by the arrow) in normoxia and hypoxia in U373MG cells transiently transfected with control or TLR9 oligo siRNA molecules. The blot was stripped and reblotted with anti-actin antibodies to demonstrate equal loading. (E) G-quadruplex-DNA (DNA) and hypoxia-induced invasion of the U373MG cells demonstrated differences in the invasive responses between control and TLR9 siRNA cells. Data are presented as a fold-change in invasion as compared with the corresponding control (as indicated by the dotted line, corresponding to vehicle treatment for G-quadruplex-DNA-induced invasion, and invasion in normoxia for the hypoxia-induced invasion) (mean ± SEM; n=7–9). ^*^P<0.01, vs. the corresponding control condition; and ^#^P<0.05 and ^##^P<0.01, vs. the control siRNA cells. TLR9, toll-like recptor-9; Nor, normoxia; Hyp, hypoxia.

Next, the involvement of TLR9 in hypoxia-induced invasion was studied more specifically, using the D54MG and U373MG control and TLR9 siRNA cells. As with the parental cells, hypoxia induced TLR9 mRNA expression in the control siRNA cell lines, as well as in the TLR9 siRNA cells. The hypoxia-induced increases in TLR9 mRNA expression were, however, significantly reduced in the TLR9 siRNA cells (Fig. 4A). As predicted, TLR9 protein expression levels were also found to decrease under hypoxic conditions in the two TLR9 siRNA cells, when compared with those of the control siRNA cells (Fig. 4B). Invasion assays were also used to study these cells; hypoxia induced an increased level of invasion in the control siRNA cells, however, invasion was evidently reduced in the TLR9 siRNA cell lines (Fig. 4C). In an attempt to achieve an improved level of TLR9 protein downregulation, TLR9 expression was also downregulated in the U373MG cells using an oligo siRNA approach. With this approach, the U373MG cells transfected with the TLR9 siRNA molecules demonstrated a complete loss of hypoxia-inducible TLR9 protein expression (Fig. 4D). Similar to the results obtained with the plasmid-based siRNA, the oligo TLR9 siRNA U373MG cells also demonstrated a significantly inhibited response to G-quadruplex-DNA and hypoxia-induced invasion, as compared with the corresponding control oligo siRNA cells (Fig. 4E). In conclusion, these results suggest that the downregulation of TLR9 expression inhibits the hypoxia-induced invasion of brain cancer cells *in vitro*.

**Figure 5:**
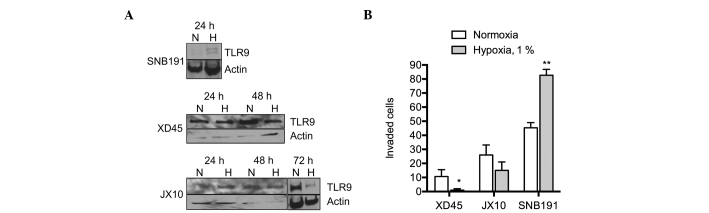
(A) Western blot analysis of TLR9 protein following culture in normoxia or hypoxia. The same blots were stripped and reblotted with anti-actin antibodies to demonstrate equal loading. (B) Cellular invasion in normoxia and hypoxia. Data are presented as the mean ± SD (n=3–6) ^*^P<0.05 and ^**^P<0.01, vs. the corresponding cells in normoxia. TLR9, toll-like receptor-9; N, normoxia; H, hypoxia.

Finally, the effect of hypoxia on TLR9 expression and invasion was investigated in other cell lines. Consistent with the previously described observations, hypoxia increased invasion in the SNB191 glioma cells, whereby the TLR9 protein was also observed to be upregulated (Fig. 5A and B). By contrast, invasion was not upregulated by hypoxia in the primary JX10 and XD45 glioma cell lines, where the protein expression of TLR9 was not found to increase, or to be downregulated by hypoxia (Fig. 5A and B). Therefore, these results suggest that TLR9 mediates hypoxia-induced invasion in brain cancer cells *in vitro*.

### TLR9 regulates MMP and TIMP-3 expression in hypoxia

**Figure 6:**
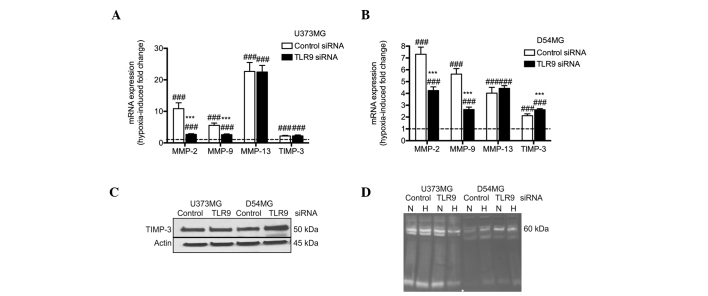
(A) U373MG and (B) D54MG control and TLR9 siRNA cells were cultured for 24 h under normoxia or hypoxia, following which the expression of the indicated mRNAs was measured using quantitative polymerase chain reaction. Data are presented as hypoxia-induced changes relative to normoxia, as indicated by the dotted line (mean ± SD; n=6). ^***^P<0.001, vs. the corresponding values in normoxia; and ^###^P<0.001, vs. the corresponding control siRNA cells. (C) Western blot analysis of TIMP-3 protein expression in the same cells following 24 h of culture in hypoxia. Actin bands were used to indicate equal loading. (D) Zymograph results from the supernatants of the same cells cultured for 24 h in normoxia or hypoxia. TLR9, toll-like receptor-9; TIMP-3, tissue inhibitor of matrix metalloproteinases-3.

Next, the mechanisms that may explain the TLR9-mediated invasion in hypoxia were investigated using control and TLR9 siRNA D54MG and U373MG cells. Firstly, the hypoxia-induced fold changes of selected mRNAs versus normoxia between the corresponding control and TLR9 siRNA cells were compared. Relative to normoxia and similar to the parental cells, hypoxia induced significant increases in MMP-2, -9 and -13 expression in the two control siRNA cells. With the exception of MMP-13 mRNA, the hypoxia-induced changes were TLR9-dependent and significantly reduced in the two TLR9 siRNA cell lines. Furthermore, similar to the parental cells, hypoxia also induced TIMP-3 mRNA expression in the two control cell lines, and this effect was significantly enhanced in the D54MG/TLR9 siRNA cells. In the U373MG/TLR9 siRNA cells, hypoxia-induced effects on TIMP-3 mRNA expression were similar to those detected in the control siRNA cells (Fig. 6A). These changes in TIMP-3 mRNA expression were also found to correlate with the TIMP-3 protein expression levels; TIMP-3 protein expression was found to be similar in U373MG/control siRNA and TLR9 siRNA cells in hypoxia. However, in the D54MG/TLR9 siRNA cells, TIMP-3 protein expression levels were increased compared with the levels observed in the corresponding control siRNA cells in hypoxia. In contrast to the normoxic conditions, the change in the TIMP-3 expression level was detected as a ~50 kDa band (Fig. 6C). Zymograms of the supernatants of the corresponding cells further indicated a decreased induction of proteolytic activity by hypoxia, particularly in the U373MG/TLR9 siRNA cells. Among the D54MG cells, hypoxia-induced proteolytic activity was observed in the control siRNA cells, but not in the corresponding TLR9 siRNA cells (Fig. 6D). Therefore, these results suggest that hypoxia-induced proteolytic and invasive activity is mediated by TLR9 in D54MG and U373MG cells, possibly via the regulation of MMPs and their endogenous inhibitors.

## Discussion

TLR9 is an innate immunity receptor, which recognizes self- and microbe-derived DNA (37,38). Although widely expressed in various tumors, including brain cancer, the contribution of TLR9 to cancer pathophysiology remains unclear (16,39) and the regulation of TLR9 expression in cancer is also poorly understood.

The present study demonstrated that the surrounding oxygen level has an important effect on TLR9 expression and function in human brain cancer cells *in vitro*. In addition, our studies further suggest that TLR9 expression is significantly associated with the invasive machinery in brain cancer cells and may mediate hypoxia-induced invasion in brain cancer. This novel observation may have therapeutic implications in brain cancers, particularly for those tumors that exhibit high hypoxia-associated TLR9 expression at diagnosis. The marginal changes in TLR9 expression observed in the TLR9 siRNA cells in normoxia, which appear to regulate MMP and TIMP-3 expression, suggest that TLR9, as a DNA-binding protein, may exhibit transcriptional activity. However, this hypothesis requires further study. Although hypoxia also induces TLR9 expression in breast cancer cells, we previously demonstrated, particularly in triple-negative breast cancers, that decreased TLR9 expression does not inhibit hypoxia-induced invasion but rather augments it. This effect was associated with a complete lack of TIMP-3 expression and was not detected in breast cancer cells that express the estrogen receptor (31). Overall, these observations suggest that, although hypoxia appears to regulate TLR9 expression in various cancer cells, the effects of TLR9 inhibition on hypoxia-induced invasion are specific to cancer type. This is to be expected, as it was recently demonstrated that TLR9 may utilize as many as 190 different cofactors to facilitate various cellular responses (40).

Hypoxic tumor regions are common in brain cancer, including GBM, and thus hypoxia is used as diagnostic criteria (41). Increased tumor hypoxia is also associated with poor prognosis in GBM, as hypoxia is important in GBM invasion (42,43). The less invasive phenotype of TLR9 siRNA cells in normoxia and hypoxia suggests that TLR9 may mediate the high levels of brain cancer cell invasion into the healthy brain tissue in clinical tumors, through regulation MMP expression and activity. Notably, hypoxia has also been shown to activate MMP-2, -9 and -13 in brain tissues; however, the current study is the first to demonstrate that these proteases may be TLR9-regulated in brain cancer cells (42,44). The results from a previous study suggesting that the absence of TLR9 expression protects against ischemia-reperfusion injury in the liver also imply that hypoxia-induced changes may be TLR9-mediated in other tissues (45). Furthermore, since the procedures of the current study were performed without the addition of exogenous ligands, the results additionally suggest that TLR9 expression is sufficient to control cancer cell invasion. However, the presence of TLR9 ligands can not be ignored, since apoptotic DNA and other endogenous ligands have been demonstrated to stimulate TLR9 expression in mammalian cells and, therefore, may have been present under the experimental circumstances (38,46). These observations add TLR9 to the list of molecules that have been previously suggested to mediate hypoxia-induced invasion, including MMP-2, Jagged-2, vascular endothelial growth factor, fibroblast growth factors and semaphorin 3F (47,48).

Hypoxia-inducible factor (HIF)-1 is a major regulator of hypoxic responses in cells, that comprises of two subunits (HIF-1α and HIF-1β), which binds to hypoxia responsive elements upstream of the hypoxia-regulated target genes (49). HIF-1α is particularly responsible for the cellular changes in acute hypoxia and, furthermore, HIF-1 expression has been detected in clinical cohorts of glioma; inhibition of HIF-1α expression reduces hypoxia-induced invasion in glioma cells *in vitro* and *in vivo*. An association has been established between HIF-1α and TLRs, as stimulation with TLR ligands has been shown to result in the accumulation of the HIF-1α protein, or to result in cellular responses that are mediated by HIF-1α (50,51). However, hypoxia has also been shown to upregulate TLR2, 4, 6 and 9 via HIF-1α (26,31,52). The reciprocal interaction between TLRs and HIF-1α suggests that hypoxia-induced TLR9 expression may also be HIF-1α-regulated in brain cancer cells. A recent study by Sinha *et al* further suggested that such regulation may also occur in states other than hypoxia (53); however, further study is required to confirm these theories.

In conclusion, hypoxia regulates TLR9 expression in brain cancer cells *in vitro* and TLR9 also mediates the hypoxia-induced invasion of these cells. Overall, these observations suggest that TLR9 expression may contribute to the increased invasion of brain cancer cells under hypoxic tissue conditions. Although this phenomenon requires further study *in vivo*, the results of the current study suggest that the suppression of TLR9 expression and activity may present a novel molecular target in brain cancer.

## Figures and Tables

**Table I:** Basal mRNA expression in the TLR9 siRNA cells relative to the corresponding control siRNA cells in normoxia.

mRNA	D54MG TLR9 siRNA	U373MG TLR9 siRNA
TLR9	0.34±0.03^c^	0.63±0.08^c^
MMP-2	1.24±0.09^b^	1.19±0.09^a^
MMP-9	0.99±0.12^d^	1.27±0.14^b^
MMP-13	1.31±0.11^b^	1.09±0.10^d^
TIMP-3	1.47±0.101^c^	1.26±0.04^c^

a P<0.05,

b P<0.01 and

c P<0.001;

d P>0.05.

TLR9, Toll-like receptor-9; MMP, matrix metalloproteinase; TIMP, tissue inhibitor of metalloproteinases.

## References

[1] Akira S, Hemmi H (2003). Recognition of pathogen-associated molecular patterns by TLR family. Immunol Lett.

[2] Wagner H (2004). The immunobiology of the TLR9 subfamily. Trends Immunol.

[3] Matsumoto M, Funami K, Tanabe M (2003). Subcellular localization of Toll-like receptor 3 in human dendritic cells. J Immunol.

[4] Nishiya T, DeFranco AL (2004). Ligand-regulated chimeric receptor approach reveals distinctive subcellular localization and signaling properties of the Toll-like receptors. J Biol Chem.

[5] Schmausser B, Andrulis M, Endrich S (2004). Expression and subcellular distribution of toll-like receptors TLR4, TLR5 and TLR9 on the gastric epithelium in *Helicobacter pylori* infection. Clin Exp Immunol.

[6] Leifer CA, Kennedy MN, Mazzoni A, Lee C, Kruhlak MJ, Segal DM (2004). TLR9 is localized in the endoplasmic reticulum prior to stimulation. J Immunol.

[7] Palm NW, Medzhitov R (2009). Pattern recognition receptors and control of adaptive immunity. Immunol Rev.

[8] Schmausser B, Andrulis M, Endrich S, Müller-Hermelink HK, Eck M (2005). Toll-like receptors TLR4, TLR5 and TLR9 on gastric carcinoma cells: an implication for interaction with *Helicobacter pylori*. Int J Med Microbiol.

[9] Schaefer TM, Desouza K, Fahey JV, Beagley KW, Wira CR (2004). Toll-like receptor (TLR) expression and TLR-mediated cytokine/chemokine production by human uterine epithelial cells. Immunology.

[10] Bowman CC, Rasley A, Tranguch SL, Marriott I (2003). Cultured astrocytes express toll-like receptors for bacterial products. Glia.

[11] Platz J, Beisswenger C, Dalpke A (2004). Microbial DNA induces a host defense reaction of human respiratory epithelial cells. J Immunol.

[12] Mempel M, Voelcker V, Köllisch G (2003). Toll-like receptor expression in human keratinocytes: nuclear factor kappaB controlled gene activation by *Staphylococcus aureus* is toll-like receptor 2 but not toll-like receptor 4 or platelet activating factor receptor dependent. J Invest Dermatol.

[13] Zaks-Zilberman M, Zaks TZ, Vogel SN (2001). Induction of proinflammatory and chemokine genes by lipopolysaccharide and paclitaxel (Taxol) in murine and human breast cancer cell lines. Cytokine.

[14] Ilvesaro JM, Merrell MA, Li L (2008). Toll-like receptor 9 mediates CpG oligonucleotide-induced cellular invasion. Mol Cancer Res.

[15] Ilvesaro JM, Merrell MA, Swain TM (2007). Toll like receptor-9 agonists stimulate prostate cancer invasion in vitro. Prostate.

[16] Merrell MA, Ilvesaro JM, Lehtonen N (2006). Toll-like receptor 9 agonists promote cellular invasion by increasing matrix metalloproteinase activity. Mol Cancer Res.

[17] Wang C, Cao S, Yan Y (2010). TLR9 expression in glioma tissues correlated to glioma progression and the prognosis of GBM patients. BMC Cancer.

[18] Jukkola-Vuorinen A, Rahko E, Vuopala KS (2009). Toll-like receptor-9 expression is inversely correlated with estrogen receptor status in breast cancer. J Innate Immun.

[19] Väisänen MR, Jukkola-Vuorinen A, Vuopala KS, Selander KS, Vaarala MH (2013). Expression of Toll-like receptor-9 is associated with poor progression-free survival in prostate cancer. Oncol Lett.

[20] Väisänen MR, Väisänen T, Jukkola-Vuorinen A (2010). Expression of toll-like receptor-9 is increased in poorly differentiated prostate tumors. Prostate.

[21] Berger R, Fiegl H, Goebel G (2010). Toll-like receptor 9 expression in breast and ovarian cancer is associated with poorly differentiated tumors. Cancer Sci.

[22] Grauer OM, Molling JW, Bennink E (2008). TLR ligands in the local treatment of established intracerebral murine gliomas. J Immunol.

[23] Zhao D, Alizadeh D, Zhang L (2011). Carbon nanotubes enhance CpG uptake and potentiate antiglioma immunity. Clin Cancer Res.

[24] Ruan K, Song G, Ouyang G (2009). Role of hypoxia in the hallmarks of human cancer. J Cell Biochem.

[25] Bar EE (2011). Glioblastoma, cancer stem cells and hypoxia. Brain Pathol.

[26] Kuhlicke J, Frick JS, Morote-Garcia JC, Rosenberger P, Eltzschig HK (2007). Hypoxia inducible factor (HIF)-1 coordinates induction of Toll-like receptors TLR2 and TLR6 during hypoxia. PLoS One.

[27] Filippova N, Yang X, King P, Nabors LB (2012). Phosphoregulation of the RNA-binding protein Hu antigen R (HuR) by Cdk5 affects centrosome function. J Biol Chem.

[28] Filippova N, Yang X, Wang Y (2011). The RNA-binding protein HuR promotes glioma growth and treatment resistance. Mol Cancer Res.

[29] Sandholm J, Kauppila JH, Pressey C (2012). Estrogen receptor-α and sex steroid hormones regulate Toll-like receptor-9 expression and invasive function in human breast cancer cells. Breast Cancer Res Treat.

[30] Merrell M, Suarez-Cuervo C, Harris KW, Väänänen HK, Selander KS (2003). Bisphosphonate induced growth inhibition of breast cancer cells is augmented by p38 inhibition. Breast Cancer Res Treat.

[31] Tuomela J, Sandholm J, Karihtala P (2012). Low TLR9 expression defines an aggressive subtype of triple-negative breast cancer. Breast Cancer Res Treat.

[32] Kauppila JH, Karttunen TJ, Saarnio J (2013). Short DNA sequences and bacterial DNA induce esophageal, gastric, and colorectal cancer cell invasion. APMIS.

[33] Muñoz-Nájar UM, Neurath KM, Vumbaca F, Claffey KP (2006). Hypoxia stimulates breast carcinoma cell invasion through MT1-MMP and MMP-2 activation. Oncogene.

[34] Arvelo F, Cotte C (2009). Hypoxia in cancer malignity. Review Invest Clin.

[35] Langton KP, Barker MD, McKie N (1998). Localization of the functional domains of human tissue inhibitor of metalloproteinases-3 and the effects of a Sorsby’s fundus dystrophy mutation. J Biol Chem.

[36] Stricklin GP (1986). Human fibroblast tissue inhibitor of metalloproteinases: glycosylation and function. Coll Relat Res.

[37] Hemmi H, Takeuchi O, Kawai T (2000). A Toll-like receptor recognizes bacterial DNA. Nature.

[38] Lande R, Gregorio J, Facchinetti V (2007). Plasmacytoid dendritic cells sense self-DNA coupled with antimicrobial peptide. Nature.

[39] Meng Y, Kujas M, Marie Y (2008). Expression of TLR9 within human glioblastoma. J Neurooncol.

[40] Chiang CY, Engel A, Opaluch AM (2012). Cofactors required for TLR7- and TLR9-dependent innate immune responses. Cell Host Microbe.

[41] Yang L, Lin C, Wang L, Guo H, Wang X (2012). Hypoxia and hypoxia-inducible factors in glioblastoma multiforme progression and therapeutic implications. Exp Cell Res.

[42] Chen W, Hartman R, Ayer R (2009). Matrix metalloproteinases inhibition provides neuroprotection against hypoxia-ischemia in the developing brain. J Neurochem.

[43] Méndez O, Zavadil J, Esencay M (2010). Knock down of HIF-1alpha in glioma cells reduces migration in vitro and invasion in vivo and impairs their ability to form tumor spheres. Mol Cancer.

[44] Lu DY, Yu WH, Yeh WL (2009). Hypoxia-induced matrix metalloproteinase-13 expression in astrocytes enhances permeability of brain endothelial cells. J Cell Physiol.

[45] Bamboat ZM, Balachandran VP, Ocuin LM, Obaid H, Plitas G, DeMatteo RP (2010). Toll-like receptor 9 inhibition confers protection from liver ischemia-reperfusion injury. Hepatology.

[46] Rifkin IR, Leadbetter EA, Busconi L, Viglianti G, Marshak-Rothstein A (2005). Toll-like receptors, endogenous ligands, and systemic autoimmune disease. Immunol Rev.

[47] Tuomela J, Grönroos TJ, Valta MP (2010). Fast growth associated with aberrant vasculature and hypoxia in fibroblast growth factor 8b (FGF8b) over-expressing PC-3 prostate tumour xenografts. BMC Cancer.

[48] Barcellos-Hoff MH, Newcomb EW, Zagzag D, Narayana A (2009). Therapeutic targets in malignant glioblastoma microenvironment. Semin Radiat Oncol.

[49] Kimbro KS, Simons JW (2006). Hypoxia-inducible factor-1 in human breast and prostate cancer. Endocr Relat Cancer.

[50] Nicholas SA, Oniku AE, Sumbayev VV (2010). Myeloid cell death associated with Toll-like receptor 7/8-mediated inflammatory response. Implication of ASK1, HIF-1 alpha, IL-1 beta and TNF-alpha. Mol Immunol.

[51] Paone A, Galli R, Gabellini C (2010). Toll-like receptor 3 regulates angiogenesis and apoptosis in prostate cancer cell lines through hypoxia-inducible factor 1 alpha. Neoplasia.

[52] Kim SY, Choi YJ, Joung SM, Lee BH, Jung YS, Lee JY Hypoxic stress up-regulates the expression of Toll-like receptor 4 in macrophages via hypoxia-inducible factor. Immunology.

[53] Sinha S, Koul N, Dixit D, Sharma V, Sen E (2011). IGF-1 induced HIF-1α-TLR9 cross talk regulates inflammatory responses in glioma. Cell Signal.

